# Nickel-adsorbed two-dimensional Nb_2_C MXene for enhanced energy storage applications†

**DOI:** 10.1039/d2ra00014h

**Published:** 2022-02-08

**Authors:** Ayesha Zaheer, Syedah Afsheen Zahra, Muhammad Z. Iqbal, Asif Mahmood, Salem Ayaz Khan, Syed Rizwan

**Affiliations:** Physics Characterization and Simulations Lab (PCSL), Department of Physics, School of Natural Sciences (SNS), National University of Sciences and Technology (NUST) Islamabad 44000 Pakistan syedrizwan@sns.nust.edu.pk syedrizwanh83@gmail.com; Department of Chemical and Petroleum Engineering, United Arab Emirates University PO Box 15551 Al-Ain United Arab Emirates mziqbal@uaeu.ac.ae; School of Chemical and Biomolecular Engineering (SCBE), The University of Sydney (USyd) Sydney Australia asif.mahmood@sydney.edu.au; New Technologies Research Centre, University of West Bohemia Univerzitni 2732 306 14 Pilsen Czech Republic sayaz_usb@yahoo.com

## Abstract

Owing to the tremendous energy storage capacity of two-dimensional transition metal carbides (MXenes), they have been efficiently utilized as a promising candidate in the field of super-capacitors. The energy storage capacity of MXenes can be further enhanced using metal dopants. Herein, we have reported the synthesis of pristine and nickel doped niobium-carbide (Nb_2_C) MXenes, their computational and electrochemical properties. Upon introduction of nickel (Ni) the TDOS increases and a continuous DOS pattern is observed which indicates coupling between Ni and pristine MXene. The alterations in the DOS, predominantly in the nearby region of the Fermi level are profitable for our electrochemical applications. Additionally, the Ni-doped sample shows a significant capacitive performance of 666.67 F g^−1^ which can be attributed to the additional active sites generated by doping with Ni. It is worth noting that doped MXenes exhibited a capacitance retention of 81% up to 10 000 cycles. The current study unveils the opportunities of using MXenes with different metal dopants and hypothesize on their performance for energy storage devices.

## Abstract 2

Owing to the tremendous energy storage capacity of two-dimensional transition metal carbides (MXenes), they have been efficiently utilized as a promising candidate in the field of super-capacitors.

## Graphical abstract

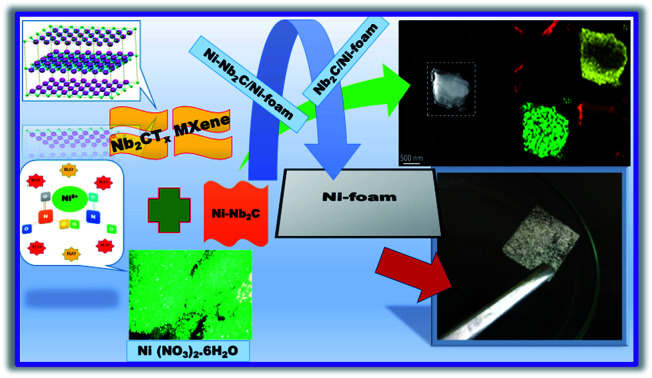

## Introduction

Energy production and storage is a hot topic of pronounced importance, and therefore, researchers across the globe have put tremendous efforts into cultivating solutions in this realm. An extensive variety of energy storage devices have been anticipated in this domain differing in storage capacity, retention, application area, charge/discharge speed, size, *etc.* Apart from batteries and several electronic memory devices, electrochemical supercapacitors are well renowned for energy storage applications.^1–3^ Supercapacitors store energy either *via* adsorption of ions (electrochemical double-layer capacitors (*i.e.* EDLCs)) or fast redox reactions at the surface (pseudo-capacitors). They can supplement or substitute batteries for high power delivery in electrical energy storage and garnering applications.^4^ The performance of any capacitor largely depends on the type of electrodes used, surface area, and type of electrolyte. Two-dimensional (2-D) materials, due to their ion-intercalation and various properties, have gained much attention for energy storage applications.^5–7^ Several new members of the 2-D materials family have been discovered such as hexagonal boron nitride,^8^ graphene,^9^ metal chalcogenides,^10^ SnO_2_,^11^ SnO_2_/SnS_2_,^12^ MoS_2_,^13^ cobalt selenide^14^ and quite a lot more. However, in electrode applications, 2-D materials show certain drawbacks such as low conductivity, narrow interlayer spacing, and hydrophobic nature.^15^ Therefore, improving the efficiency of electrodes by exploring new and innovative electrode materials^16^ is a future endeavor in both academic and industrial research. In 2011 Gogotsi and his team, at Drexel University, USA, reported a new 2-D material called MXene.^17^ MXenes possess unique features of well-defined geometry, fast ion/molecule diffusion, excellent conductivity, large surface area, and hydrophilic nature which make them competent and promising electrode materials for supercapacitor. MXenes being the latest and largest addition in the family of the 2-D materials include the carbides, nitrides, and carbonitrides of transition metals.^18^ Their parent MAX phase are ductile ceramics having the general formula: M_*n*+1_AX_*n*_, where *n* ranges between 1 to 3 (*i.e.* M_2_AX, M_3_AX_2_, or M_4_AX_3_, *etc.*); M denotes transition metals (*e.g.* Ti, Nb, V, Mo, W, Ta); A can be an element from group III-A or IV-A (*e.g.* Al, Si, Ga); and X corresponds to atoms like carbon (for carbides), nitrogen (for nitrides) or both (carbonitrides C/N). MXenes are synthesized by carefully etching out A-element layers from the parent MAX phase while keeping a gentle balance between etching conditions.^19^ Remarkably, the exfoliated MXenes always result in terminal functional groups such as F, OH, and/or O groups,^20^ represented by M_*n*+1_X_*n*_T_*x*_. Here, T characterizes surface terminations (–OH, =O, and/or –F) and *x* represents the number of termination groups for each formula unit.^21^ The van der Waals forces of attraction are considered responsible for holding the structures in graphite and other layered materials, whereas ionic, covalent, and metallic bonds are responsible for unique structures in MAX. The bonding between M and X is directionally covalent and strong, and the bond between M and A is comparatively feebler than that between M and X.^22,23^ Due to this relative difference in bond strengths, A layer (*i.e.*, Al/Si) can be selectively etched-out without disrupting the original structure.

Doping of foreign elements in 2-D materials acquaint them with impurities to impart desirable features aimed specifically at targeted applications. Doping of MXenes causes an increase in surface area and *c*-lattice parameter due to defects and impurities by decreasing particle size in order to improve their properties for energy storage applications.^24^ The introduction of metal dopant in MXene layers not only increases specific surface area but also alters the adsorption energetics of surface^25^In the past few years, the performance of MXenes has been greatly enhanced for enormous applications by modification in their surface chemistry and/or structural modification either through doping or hybrid formation. Sundus *et al.* doped Nb_2_C MXene with erbium (Er) for electrochemical hydrazine sensing.^26^ Similarly, Jameela *et al.* reported magnetic transition behavior in lanthanum (La) doped Nb_2_C MXene.^27^ Likewise Chenyang *et al.* reported the synthesis of Ni(OH)_2_/Ti_3_C_2_ composite for lithium ion battery.^28^ MXene/Ni chain hybrid (Liang *et al.*, 2019) has shown excellent electromagnetic wave absorption and shielding capacity as well. In addition to this, the doping metals in MXene also include Gd,^29^ Co,^25^ Cr,^30^ Fe_3_O_4_,^31^ MnO_2_.^32^ In comparison with other, Ni possessed high electrochemical behavior because of their better electronic conductivity and rich redox reactions and has been explored widely in different materials.^33^ Moreover, the size of the ionic radii of niobium Nb^2+^ (0.08 nm) is almost comparable to the ionic radii of nickel Ni^2+^ (0.07 nm); thus, making nickel an appropriate candidate to be doped in Nb_2_C MXene. On the other hand, liquid electrolytes have been utilized extensively in supercapacitors and batteries. However, gel-polymer electrolytes (GPEs) are now being explored widely due to high conductivity and electrochemical stability with almost negligible liquid contents.^34^ Among various GPEs, poly-vinyl alcohol (PVA) is the most probable GPE due to its producibility, chemical resistance, and excellent hydrophilicity.

Herein, we report structural and computational perspectives of nickel (Ni) doping in Nb_2_C MXenes. Using polymer-based electrolyte (PVA–H_2_SO_4_) rendered excellent conductivity of 220 mS and sanctioned quicker and easier surface redox reactions leading to pseudo-capacitance. In addition, the current work utilizes MXene modified Ni-foam electrode in contrast to conventional MXene paper for electrochemical measurements, which is the first reported effort of its kind. Moreover, computational results are in excellent agreement with experimental findings which present a better understanding of the proposed material system. The current study strongly anticipates in opening vast opportunities of using Nb_2_CT_*x*_ MXene along with different metal dopants, as a potential electrode material, for pseudo-capacitive energy storage technologies.

## Experimental section

### Preparation of Nb_2_CT_*x*_ MXene

2-D MXene sheets were prepared from the Nb_2_AlC MAX phase through the already optimized wet chemical etching method. 1 g of Nb_2_AlC MAX powder (200 mesh size) was immersed in 10 mL of 50% wt HF in a Teflon beaker kept under continuous magnetic stirring for ∼30 hours at 55 °C. The resulting deposits were washed several times with deionized water *via* centrifugation for 5 minutes at 3500 rpm. The washing cycles were repeated until the pH of the supernatant became ∼6.0. The resulting MXene powder was dried in a vacuum oven at 40 °C for 24 hours.^35^

### Preparation of Ni–Nb_2_CT_*x*_ MXene

**Fig. 1:**
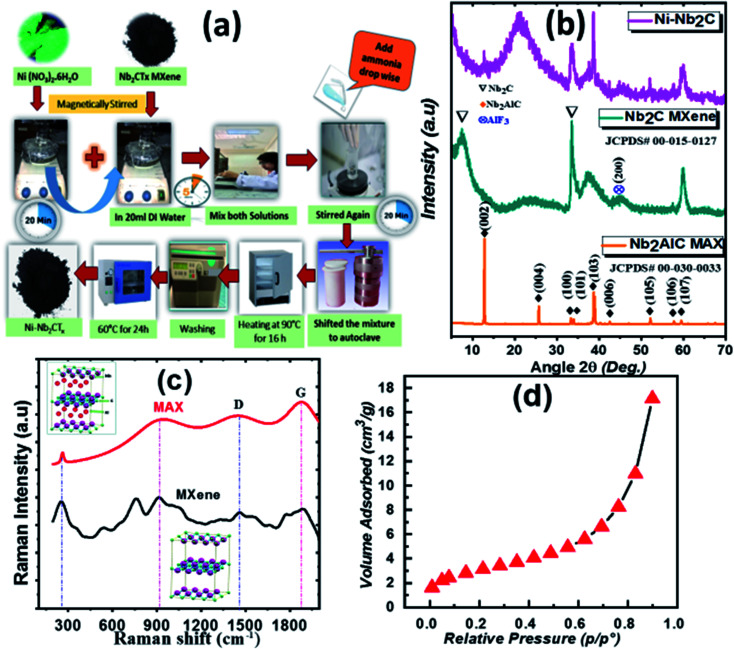
(a) Pictorial diagram for hydrothermal synthesis of Ni-doped MXene, (b) XRD patterns of MAX, MXene and, 5% Ni-doped MXene, (c) Raman spectra of MAX and MXene, and (d) N_2_ adsorption isotherm curve for Ni–Nb_2_CT_*x*_ MXene.

Nickel-doped Nb_2_C MXene with different ratios of Ni (2.5–10% by weight) (Fig. 1(a)) was synthesized by following a facile sequential hydrothermal method.^36^ The doping amount of Ni in Nb_2_C is signified in terms of different atomic weights originally taken as a sample. Specifically, black Nb_2_CT_*x*_ powder was dissolved in 30 mL deionized water under magnetic stirring for 30 minutes. The precursor solution was prepared by dissolving crystals of nickel nitrate hexa-hydrate Ni(NO_3_)_2_·6H_2_O in 20 mL deionized water. Both prepared solutions were mixed and magnetically stirred for 20 minutes. Meanwhile, 50% ammonia was added to the solution dropwise until the pH reaches 9. It was then shifted to a Teflon-lined autoclave made of stainless steel (70 mL) at 90 °C for 16 hours. Afterward, the reaction mixture was cooled to room temperature followed by washing precipitates with DI water. The resulting product was dried in a convection oven at 60 °C for 24 hours. The procedure was repeated to dope MXene with various concentrations of nickel.

### Material characterizations

Diffraction measurements were performed *via* powder X-ray diffractometer (XRD, Bruker, D8 Advance) using monochromatic Cu-Kα radiation wavelength of *λ* = 1.5405 nm under voltage source of 40 kV with 2*θ* ranging from 5° to 70°. The morphology of both, as-prepared and doped samples, was studied by scanning electron microscopy (SEM) under different resolution ranges of 2 μm, 5 μm, and 500 nm. Transmission electron microscopy (TEM) analysis was performed to understand morphology and structures of Ni–Nb_2_C MXene. High resolution TEM images were acquired on Titan 60-300 (Thermo Fischer Scientific) equipped with an imaging Cs-corrector and working at 300 kV. Elementary mappings were performed in scanning TEM (STEM) mode associated with energy-dispersive X-ray spectroscopy (EDS) detector (EDAX) and electron energy loss spectroscopy (EELS) detector (Gatan). The samples were dispersed in isopropanol and deposited on a carbon coated copper grid, followed by drying at room temperature. The chemical structure was analyzed by Fourier transform infrared spectrometer with attenuated total reflection (FTIR-ATR) from Bruker Alpha. The samples were scanned in a frequency range of 500–4000 cm^−1^.

Textural properties were studied by a micrometric gas adsorption analyzer based on Brunauer–Emmett–Teller (BET) method. Electrochemical performance of the samples was investigated using cyclic voltammetry (CV) Interface 1010B Potentiostat (Gamry). Conductance test, for the electrolytes, was performed using Jenway 4510 conductivity meter supplied with glass conductivity probe. The electrochemical performance of pristine MXene and nickel doped MXene samples were examined by using three electrode organization with Ag/AgCl as reference electrode, platinum as a counter electrode, and Ni-foam electrode used as working electrode in 1 M gel PVA–H_2_SO_4_ electrolyte at different scan rates in the potential window range of −0.2 V to –0.4 V. To study the theoretical properties of pristine Nb_2_CT_*x*_ and Ni-doped Nb_2_CT_*x*_ (T_*x*_ characterizes surface terminations like –OH and/or =O, –F so we have oxygen (O) and fluorine (F)), density functional theory (DFT) was employed in this work. For DFT, all the calculations were executed using full potential linearized augmented plane wave (FLAPW) utilizing the framework of Wien2k code.^37^ The exchange–correlation potential was solved using Perdew–Burke–Ernzerhof (PBE) generalized gradient^35^ approximation (PBE-GGA) functional.^38^

## Results and discussion

### X-ray diffraction

X-ray diffraction patterns of MAX, MXene and, Ni-doped MXene, in the detection range of 5–70°, are shown in Fig. 1(b). The diffraction peaks in MAX are indexed following JCPDF: 00-030-0033, showing hexagonal *P*6_3_/*mmc* symmetry.^35^ The peak in MAX at 2*θ* ∼ 38.9° vanished after HF treatment which is attributed to removal of Al layer from the MAX phase. Meanwhile, the (002) peak is broadened and shifted from 12.792° (in MAX) to 9.302° (in Nb_2_CT_*x*_ MXene) after etching treatment with an increase in *c*-lattice parameter (*c*-LP) from 13.83 Å to 20.73 Å, suggesting an enhanced interlayer spacing in MXene.^39^ The diffraction pattern observed in Ni-doped Nb_2_C MXene exhibited decent matching with pristine Nb_2_C MXene.^40^ The characteristic (002) peak of Ni–Nb_2_CT_*x*_ was shifted to a lower angle, indicating successful expansion of interlayer spacing attributable to intercalation caused by nickel. The XRD data of Ni-doped MXene at other concentrations is also shown in Fig. S1† in which, intensity of peaks at 2*θ* ∼ 33° and 2*θ* ∼ 38.6° corresponding to (100) and (110) planes, decreased with increasing Ni concentration indicating a subsequent decrease in particle size, attributed to loss of crystallinity due to lattice distortion.^41^ The ionic radius of Nb^2+^ (0.08 nm) is comparable to that of Ni^2+^ (0.07 nm), indicating two possibilities that may arise as a result of doping: (1) Ni substituted Nb in MXene, or (2) Ni accumulation at the boundary interacting chemically with surface terminations. Moreover, when Ni^2+^ ions are incorporated into the hexagonal crystal lattice of Nb_2_CT_*x*_, an electrostatic potential is developed between Ni cation and negatively charged surface terminations, causing a stress in the system that may lead to a decrease in particle size.^29^

### Raman spectroscopic analysis

The defects in the crystal structure of MAX, and MXene were studied by Raman spectroscopy. The Raman spectrum of MAX and MXene (Fig. 1(c)) exhibited several vibrational modes in the range of 200–2000 cm^−1^. Majority of the peaks are broadened and shifted to higher wavenumbers in MXene compared to MAX, indicating bond strengthening between Nb–C atoms.^42^ The first peak (∼250 cm^−1^) in MAX was broadened and became less intense after etching treatment, indicating either the removal of Al or exchange of Al with some lighter atoms such as O, F, N.^43^ The 2^nd^ peak at 920 cm^−1^, associated with C, was also broadened and downshifted.^44,45^ The presence of C in the Raman spectrum suggests that sharper and intense peaks are most likely due to Nb carbide ordered phase.^46^ The last two peaks in Raman spectra represent the D- and G-bands. The D-band (disordered carbon) is produced due to sp^3^ hybridized carbon, whereas the G-band is a signature of sp^2^ carbon structure arising due to E_2g_ phononic mode at *Γ*-point of the Brillouin zone (showing C–C bond stretching). The relative intensity ratio of D to G band (*I*_D_/*I*_G_) indicates disorderliness in the crystal structure.^47,48^ Herein, the *I*_D_/*I*_G_ ratio for MAX was 0.96 that decreased to 0.93 in MXene, indicating a less defective MXene structure.^49^

### Surface area analysis

The N_2_ adsorption isotherms of Ni-doped Nb_2_CT_*x*_ (Ni = 5%) are shown in Fig. 1(d). All other isotherms (Ni = 2.5%, 7.5%, 10%) are shown in Fig. S4.† According to the classification of the International Union of Pure and Applied Chemistry (IUPAC), all isotherms display type-II behavior which demonstrates a continuous increase in adsorbed volume with pressure.^50^ The surface area of Ni–Nb_2_CT_*x*_ is 18.02 m^2^ g^−1^ as determined by the BET method. On the other hand, undoped Nb_2_CT_*x*_ MXene exhibited a surface area of about 5.21 m^2^ g^−1^. This large increase in the surface area with Ni-doping is attributed to the extended interlayer spacing with nickel ions. The increased surface area is advantageous for improving electrochemical performance through ion diffusion enhancement with more active sites present during electrochemical reaction progressions.^51^ Table S1† presents the BET surface area (m^2^ g^−1^), Langmuir surface area (m^2^ g^−1^), and particle size (Å) as a function of Ni concentration in MXene.

### Morphological analysis

**Fig. 2:**
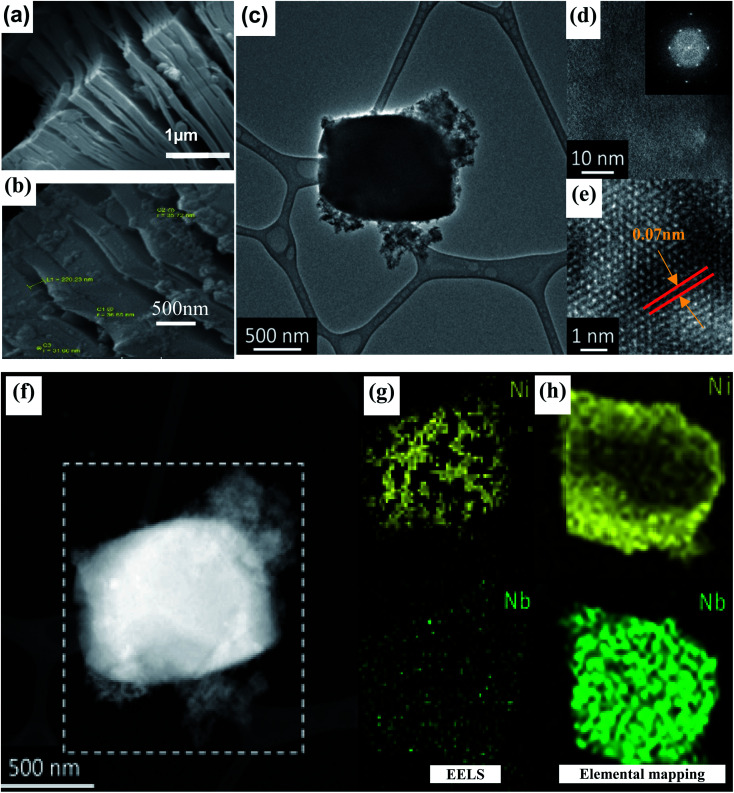
(a and b) SEM micrographs of Nb_2_CT_*x*_ MXene & Ni–Nb_2_CT_*x*_ MXene, respectively, (c) TEM images of Ni–Nb_2_CT_*x*_ MXene show a particle fabricated at low magnification (d) and (e) show high resolution images of the periphery of the same particle; the inset in (d) is the fast Fourier transform of the image indicating the hexagonal symmetry of the sample, (f–h) elemental mapping of Ni-doped Nb_2_CT_*x*_ MXene.

Surface morphology of Nb_2_CT_*x*_ and Ni–Nb_2_CT_*x*_ were observed by SEM (Fig. 2(a and b)). After etching of Al, as-synthesized Nb_2_CT_*x*_ showed layered morphology (Fig. 2(a)) similar to exfoliated graphite exhibiting distinct sheet splitting.^32^ Similarly, Ni–Nb_2_CT_*x*_ showed layered morphology with fine slanted nano-sheets (Fig. 2(b)). In addition, HRTEM results of 5% Ni-doped MXene are also shown in Fig. 2(c) (at low magnification) which indicate the existence of a thick rectangular crystal surrounded by flakes of Ni–Nb_2_C MXene. Fig. 2(d) shows the flakes at higher magnification, and inset of (d) contains fast Fourier transform of the image confirming that the resulting doped sheets have retained hexagonal symmetry of MXene.^52,53^ Moreover, Fig. 2(e) displays a vibrant crossed fringe associated with crumbled and folded edge thus, depicting the enigmatic flexibility of MXene.^54^

The elemental composition was further confirmed by EDS and EELS (Fig. 2(f–h)). EELS allows applying “white lines” analysis of elemental edges attributed to unoccupied density of state (DOS) whereas the EDS enables generating the elemental mapping.^55^ Since Nb belongs to the second row of transition elements and has numerous unoccupied d-states just above the Fermi level, it gives white lines on L3-edge.^56^ The signal for Nb is relatively low which can be explained by L3-edge location at high energy loss (2371 eV) linked to unoccupied states in the 4d-bands of Nb^57^ and a relatively thick sample responsible for intense noise signal. To overcome this difficulty, complementary EDS analysis was performed showing that Nb and Ni are distributed uniformly over the whole sample. The complete mapping of all other elements is provided in Fig. S2 and S3.†

### Computational analysis

**Fig. 3:**
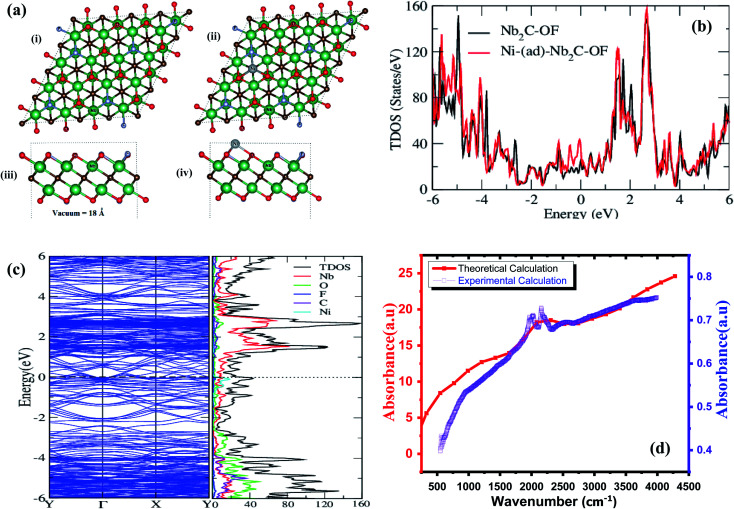
(a) (i) Structure of Nb_2_C–OF, (ii) structure of Ni adsorbed Nb_2_C–OF, (iii) the side-view of Nb_2_C–OF, (iv) the side-view of Ni adsorbed Nb_2_C–OF, (b) total density of state (TDOS) for Nb_2_C–OF and Ni adsorbed Nb_2_C–OF, (c) (left) total density of states of Ni–(ad)Nb_2_C–OF in unit cell and individual atoms in a cell + band structure (right), (d) experimental and theoretical optical properties of doped MXene.

For computational analysis, primarily, lattice parameters of Nb_2_C MXene were taken from the experimental XRD data. The structure is modeled using 4 × 4 × 1 supercell producing 18 Å of vacuum between the slabs. The crystal structure, shown in Fig. 3a(i) and (iii), is a layered hexagonal structure with space group *P*6_3_/*mmc* and lattice parameters *a* = 12.47 Å, *c* = 22.59 Å. In Fig. 3a(ii) and (iv), Ni is adsorbed on C atom at stable positions. The muffin-tin radii (*R*_MT_) were taken as 1.92, 1.78, 1.82, 1.73, 2.10 a.u. (atomic units) for Nb, O, F, C, and Ni, respectively. In the interstitial region, plane wave cutoff was calculated as the product of *R*_MT_*K*_MAX_, where *R*_MT_ is the smallest atomic sphere radius and *K*_MAX_ is the magnitude of the largest wave vector.^58^ In this work, *R*_MT_*K*_MAX_ value was 7.0, and convergence criterion for the charge and energy was chosen up to 1 × 10^−4^ Ry and 1 × 10^−3^ Ry. The internal geometry of the structure was optimized using 2 *k* points in an irreducible Brillouin zone (IBZ) of 2 × 2 × 1 while the self-consistency was achieved with 6 × 6 × 3 *k*-mesh using 54 *k* points in IBZ.

The density of states (DOS) provides an understanding of how numerous states are being occupied by various atoms and gives an insight into their valence shells at various energy intervals. Fig. 3(c) shows the total density of states (TDOS) *versus* energy (eV) plot for Nb_2_C–OF and Ni adsorbed Nb_2_C–OF. TDOS/eV for Nb_2_C–OF has a continuous spread all over the region from valence band to conduction band with enough peak height. When a certain foreign material is doped in a system, the orbital density of the dopant is most likely to come at the Fermi level. Björkman *et al.* reported similar work of doping with increased TDOS and electrical conductivity after doping was introduced.^47^ In the present report, when Ni is adsorbed (doped) at one of the Nb sites, TDOS of Ni–Nb_2_C–OF seems to get coupled with pristine MXene, showing a continuous DOS pattern. Moreover, Ni doping increased TDOS, specifically around the Fermi level with a maximum peak of Ni adsorbed–Nb_2_C–OF appearing at around 3 eV with 160 DOS/eV. To the best of our knowledge, there is no earlier data available on theoretical calculation of Ni-doped Nb_2_CT_*x*_ MXene for comparison. However, comparison with Nb doped MXene is used here to justify that Ni doping into MXene would increase DOS/eV as compared to undoped MXene.^48^ The alterations in DOS, predominantly in the nearby region of the Fermi level, would likely bring about noticeable changes in the resultant electronic properties, profitable for electrochemical applications.

The band structure of pristine MXene shows a metallic behavior as the bandgap is signified to be zero.^59–61^ After doping with a metal atom (Ni), a predominant increase in the density of electrons around the Fermi-level is observed. Here, band structures of Ni-adsorbed Nb_2_C–OF were calculated alongside high-symmetry directions *Y* → *Γ* → *X* → *Y* of irreducible Brillouin zone (BZ), and the Fermi energy level was fixed at 0 eV. The bandgap of Ni-doped structure is almost zero. Fig. 3(c) shows that the valence band maximum (VBM) and conduction band minimum (CBM) lie at the same *Γ*-point, indicating the presence of a direct bandgap. The band structure calculations show a significant spread of Nb and Ni all over the Fermi level that is attributed to the manifestation of bonding interactions arising from d-orbitals transitions. The partly occupied states of Nb, and Ni, as well as p-orbitals in C, are main contributors toward electronic density around the Fermi energy; these states cross the Fermi level, leading to metallicity in the current model.^62^

Fig. 3(c) (right panel) represents different atomic contributions in Ni-doped Nb_2_C–OF towards DOS; F showing a negligible contribution whereas Nb shows a significant contribution towards TDOS since the electronic configuration of Nb is [Kr] 4d^4^5s^1^. It not only offers the d-orbital contribution but also the s-orbital contribution towards TDOS. The DOS of C merges with DOS of Nb because the s-orbital of C is contributing towards VBM, whereas the p-orbital is contributing to the valence as well as conduction bands. Lastly, Ni has main contribution at the Fermi level because of its 8 electrons in the d-shells, out of which, 6 electrons are paired, and rest are unpaired. This observable hybridization arises from 4d-Nb, 3d-Ni, and 2p-C states in Ni-doped MXenes.

In Wien2k, the optics can be calculated with an independent particle approximation that calculates the direct transitions between occupied *nk* and unoccupied *n*′*k* states using Kohn–Sham eigenvalues.^63^ The imaginary part of dielectric function is calculated by modifying the joint density of states for transition probabilities that is given by the square of momentum matrix elements *M* = 〈*n*′*k*|*A*·*p*|*nk*〉 between these states and then dipole selection rules are used to determine the intensity of optical spectra.^64^ The real part ‘*ε*_1_’ can be obtained from imaginary part ‘*ε*_2_’ using the Kramers–Kronig transformation. The absorption coefficient (*I*) is calculated using *ε*_1_ and *ε*_2_. Our calculated *I* agrees with experimental spectra as shown in Fig. 3(d). The experimental description of FTIR data is given in Fig. S5.†

### Electrochemical analysis

**Fig. 4:**
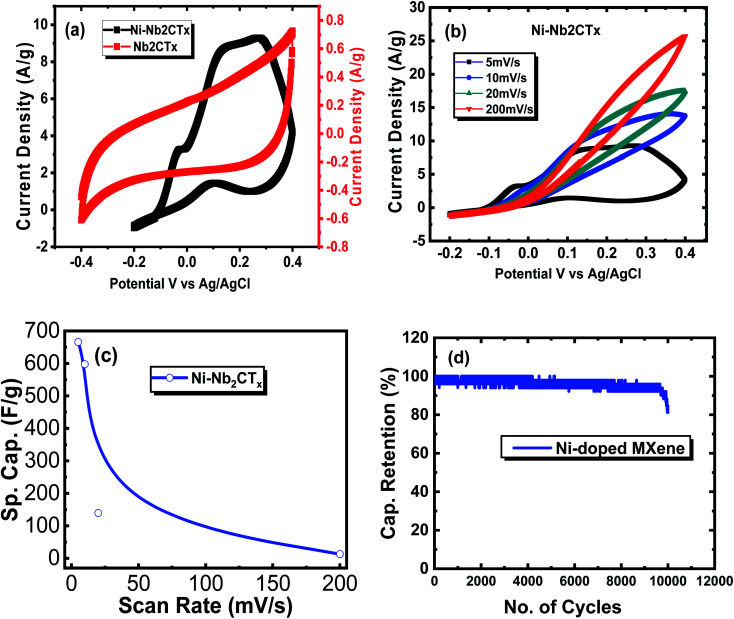
(a) Cyclic voltammogram (CV) curves for Nb_2_CT_*x*_ and Ni–Nb_2_CT_*x*_ in 1 M PVA–H_2_SO_4_ at a scan rate of 5 mV s^−1^ (b) CV curves explicitly for Ni–Nb_2_CT_*x*_ in at different scan rates (c) specific capacitance *vs.* scan rate for Ni–Nb_2_CT_*x*_ (d) capacitance retention *vs.* cycle number for Ni–Nb_2_CT_*x*_.

Electrochemical performance of pristine and Ni–Nb_2_CT_*x*_–MXenes, using a Ni-foam (NF) electrode as the working electrode in three electrode assembly, is shown in Fig. 4. Herein, pristine and Ni-doped MXenes were coated over NF working area by casting and drying a drop of MXene/ethanol suspension. The data for cyclic voltammetry were recorded with varying scan rates and symmetric *I*–*V* curves were obtained in which *y*-axis was represented as current density *i.e.* ampere per gram (A g^−1^). Fig. 4(a) is showing the cyclic voltammogram profiles of both the Nb_2_CT_*x*_ and Ni–Nb_2_CT_*x*_ at 5 mV s^−1^ in polymer gel electrolyte (poly-vinyl alcohol), PVA in H_2_SO_4_. The CV curves of the Ni–Nb_2_CT_*x*_ at different scan rates (5, 10, 20, 200 mV s^−1^) are presented in Fig. 4(b). Explicitly, Ni–Nb_2_CT_*x*_ exhibits a broad CV curve and an enhanced current density of 9.0 A g^−1^ in PVA–H_2_SO_4_ at 5 mV s^−1^, indicating a higher ionic conductivity of Ni-doped MXene.^65,66^ The gel electrolytes usually have fewer demerits compared to aqueous electrolytes for electrochemical energy storage applications. Extensive research has been carried out using polymer gel electrolyte among which PVA is more striking because of its wider voltage window, mechanical and structural stability; the presence of –OH groups in PVA are responsible for absorbing large contents of water and enhancing its ionic conductivity.^67–69^ The redox peaks were observed for all samples exhibiting pseudocapacitive behavior which indicates that there is redox transition occurring between different valence states. This increase in capacitance might be attributed to the small particle size and increased surface area, created upon doping with Ni in MXene, which would lead to additional electro-active sites increasing the capacitance subsequently. After doping, there is a predominant increase in the density of electrons around the Fermi-level which is a manifestation of bonding interactions arising from d-orbitals transitions. These alterations in DOS in the nearby region of the Fermi level bring about noticeable changes in the resultant electronic properties, which increases the metallicity that is profitable for electrochemical applications. Such findings were predicted from the DFT DOS calculations discussed in the preceding section (Table 1).

**Table 1:** Comparison of specific capacitance of two-dimensional MXenes

Material	Specific capacitance	Electrolyte	Ref.
Ti_3_C_2_ MXene (2016)	124 F g^−1^	KOH	70
Mo_2_CT_*x*_	190 F g^−1^	H_2_SO_4_	71
d-Ti_3_C_2_ (delaminated layers of Ti_3_C_2_)(2014)	320 F g^−1^	H_2_SO_4_	72
Orthorhombic niobium pentoxide (T–Nb_2_O_5_)	330 F g^−1^	H_2_SO_4_	73
Nb-doped MXene (Ti_3_C_2_) (2020)	442.7 F g^−1^	6 M KOH	74
Ni-doped Nb_2_C MXene	666.67 F g^−1^	1 M PVA–H_2_SO_4_	This work

The charge storage capacity is estimated by the capacitance (Farads, F) of a material. Specific capacitance (F g^−1^) is a function of capability of carbon to adsorb ions from an electrolyte in carbon-based supercapacitors,^75^ which is calculated as follows:^76^
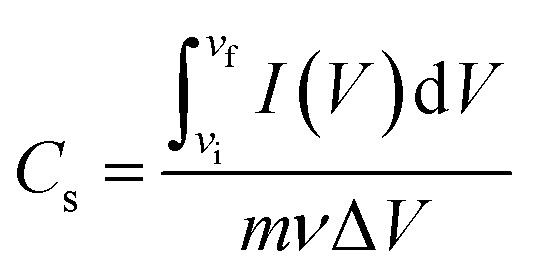
Here, *I*(*V*)d*V* is the integrated area of the *C*–*V* curve (A.V.), *m* is mass of the electroactive materials (g), *ν* is the scan rate (V s^−1^), Δ*V* is the potential difference window (V), and *v*_i_ and *v*_f_ are the voltage limits.

Fig. 4(c) shows specific capacitances *vs.* scan rates for pristine and Ni-doped MXenes. The specific capacitance decreases with increasing scan rate since the electrolyte has better contacts with the electrode material at a lower scan rate and the electrolyte completely perforates in the pores of the electrodes resulting in enhanced charge storage on the electrode surface, consequently resulting in an increased capacitance.^77^ It is to be noted that specific capacitance of 258.6 F g^−1^ was observed for pristine MXene whereas our metal modified MXene showed a significant capacitance of 666.67 F g^−1^ at 5 mV s^−1^ in PVA–H_2_SO_4_ electrolyte and this high specific capacitance of Ni-doped MXene was also predicted in our DFT analysis presented in the preceding section and is attributed to the modified electronic structure and creation of extra density of states around the Fermi level.

The cyclic stability of the electrodes is shown in Fig. 4(d). The Ni-doped MXene exhibited capacitance retention of ∼81% over 10 000 cycles. Thus, Ni-doping has enhanced the specific surface area and resultantly disclosed improved electrical conduction contributing towards better electrochemical capacitors and giving us worthy reasons to expect further improvements in capacitance of MXene with different metal dopant in different electrolytes.

## Conclusions

We have successfully synthesized two-dimensional Nb_2_CT_*x*_ MXene by etching Al-phase out and doping the etched MXene with varying Ni concentrations using a facile and effective hydrothermal technique. The structure and morphology of pristine and doped MXenes were thoroughly characterized. The EELS and EDS elemental mapping confirmed homogenous doping of Ni in MXene. Approximately, 350% increased surface area of the doped MXene is attributed to homogenous distribution of Ni on the MXene surface. The Ni-doping increased the *c*-lattice parameter of MXene from 20 Å to 22 Å as confirmed by XRD and increased DOS per eV around the Fermi level as confirmed by the DFT calculations. The MXenes were deposited over Ni-foam electrode and electrochemical properties were studied *via* cyclic voltammetry (CV). The increased current density and capacitance were displayed by Ni–Nb_2_CT_*x*_ which were attributed to additional active sites generated by Ni-doping, consequently enhancing accessibility of Nb_2_CT_*x*_ layers to electrolyte ions. The cyclic stability revealed that Ni–Nb_2_CT_*x*_ MXene could withstand ∼10 000 cycles with capacitance retention of 81%. Overall, Ni-doped Nb_2_CT_*x*_ MXene in PVA–H_2_SO_4_ electrolyte exhibited excellent conductivity and sanctioned quicker and easier surface redox reactions leading to pseudo-capacitance. This effort opens the opportunities of using MXene with different metal dopants in various electrolytes suitable for energy storage devices.

## Data availability

The data that support the findings of this study are available from the corresponding author (S. Rizwan) upon request.

## Author contribution

Ayesha Zaheer and Syedah Afsheen Zahra performed experimentation and paper writing; Muhammad Iqbal helped in HRTEM, EDX and EELS measurement analysis; Asif Mahmood helped in paper writing & analysis, Salem Ayaz Khan performed computational analysis; and Syed Rizwan conceived the scientific idea, secured financial support, helped in paper writing and supervised the complete project.

## Conflicts of interest

The authors declare that there are no competing interests.

## Supplementary Material

RA-012-D2RA00014H-s001
