# Supplementary material for: Nickel-adsorbed two-dimensional Nb2C MXene for enhanced energy storage applications
Source: RSC Adv. 2022 Feb 8;12(8):4624–34. doi: 10.1039/d2ra00014h (PMC8981252; doi:10.1039/d2ra00014h)
Supplement: RA-012-D2RA00014H-s001 [file RA-012-D2RA00014H-s001.pdf]

Electronic Supplementary Material (ESI) for RSC Advances.
This journal is © The Royal Society of Chemistry 2022

Supplementary materials

Nickel-adsorbed Two-dimensional Nb2C MXene for Enhanced Energy Storage Applications

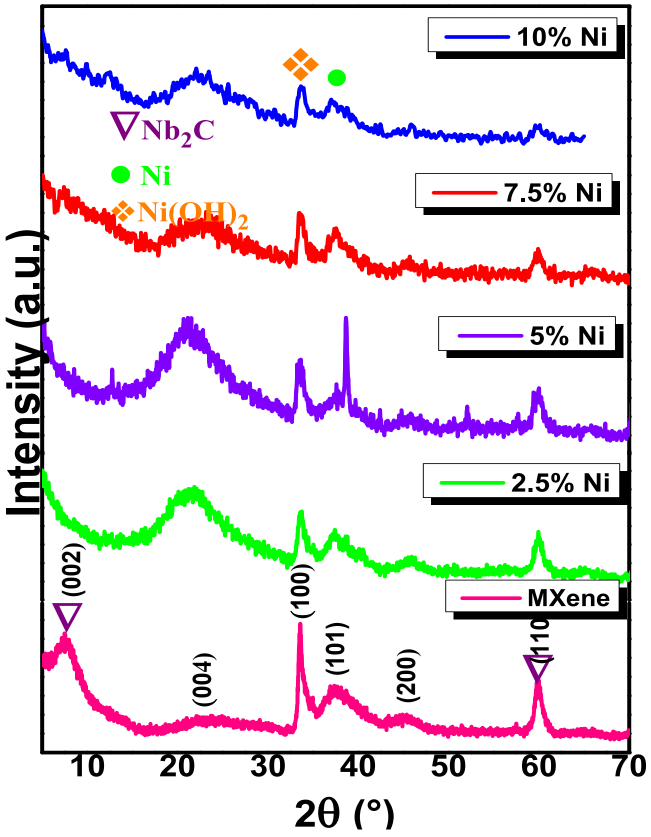

Figure S1: XRD patterns for MXene and Ni doped MXene

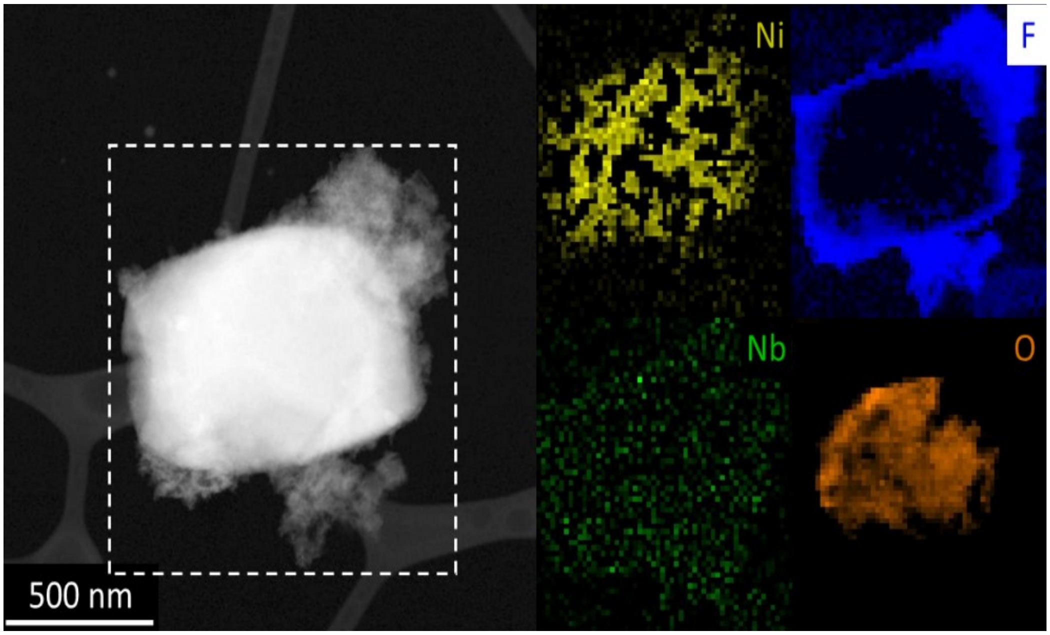

Figure S2: STEM-EELS mapping of Nb_2CTx MXene

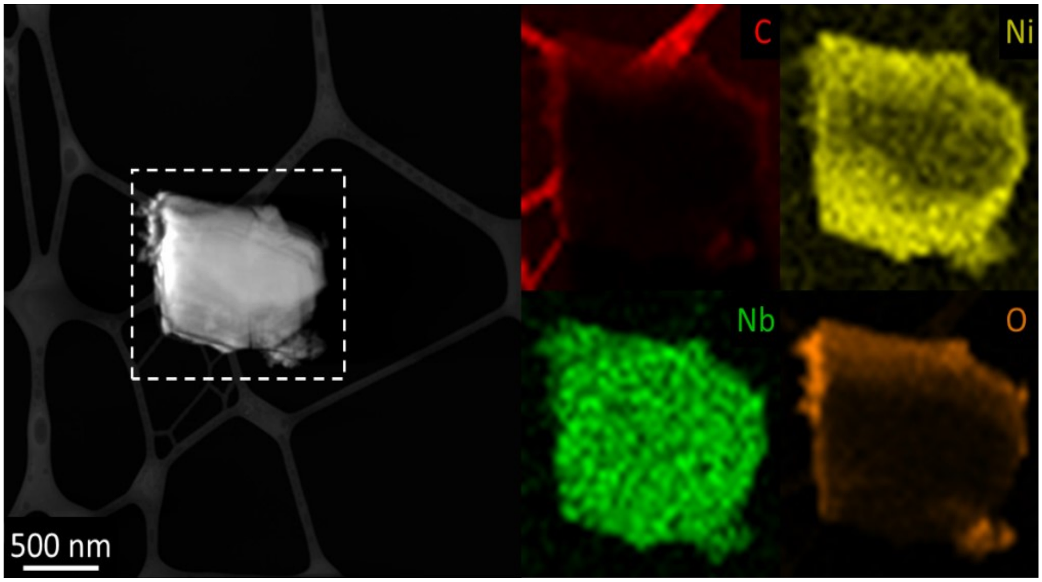

Figure S3: STEM-EDS mapping of Nb_2CTx MXene

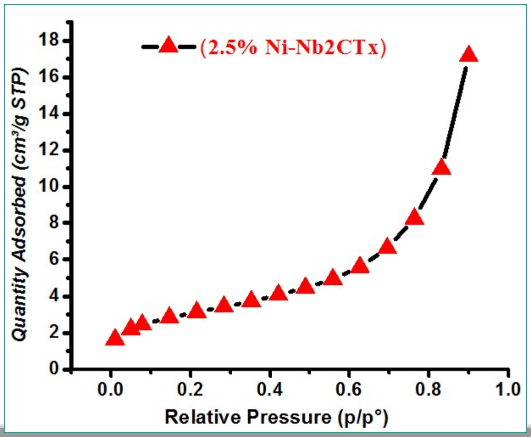
(2.5% Ni-Nb2CTx)
Quantity Adsorbed (cm³/g STP)
Relative Pressure (p/p°)
0
2
4
6
8
10
12
14
16
18
0.0
0.2
0.4
0.6
0.8
1.0

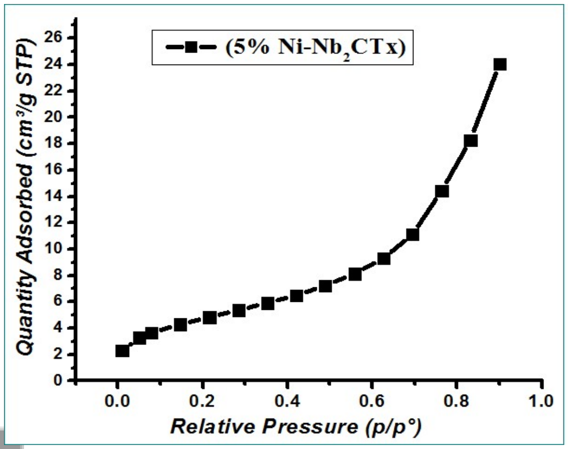
(5% Ni-Nb2CTx)
Quantity Adsorbed (cm³/g STP)
Relative Pressure (p/p°)
0
2
4
6
8
10
12
14
16
18
20
22
24
26
0.0
0.2
0.4
0.6
0.8
1.0

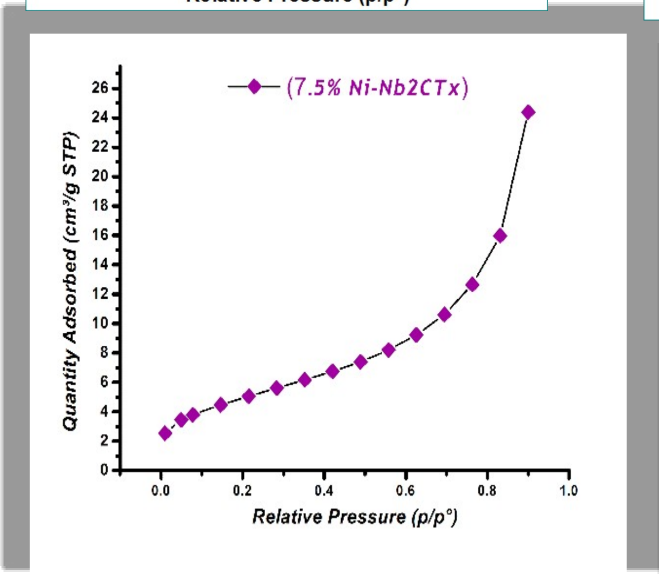
(7.5% Ni-Nb2CTx)
Quantity Adsorbed (cm³/g STP)
Relative Pressure (p/p°)
0
2
4
6
8
10
12
14
16
18
20
22
24
26
0.0
0.2
0.4
0.6
0.8
1.0

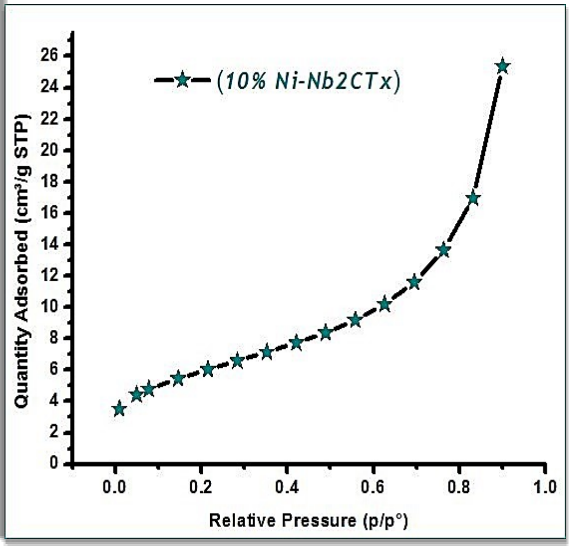
(10% Ni-Nb2CTx)
Quantity Adsorbed (cm³/g STP)
Relative Pressure (p/p°)
0
2
4
6
8
10
12
14
16
18
20
22
24
26
0.0
0.2
0.4
0.6
0.8
1.0

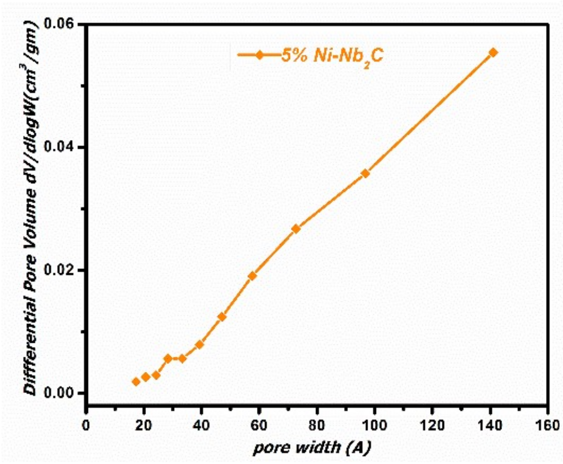
5% Ni-Nb2C
Differential Pore Volume dV/dlogW(cm³/gm)
pore width (A)
0.00
0.02
0.04
0.06
0
20
40
60
80
100
120
140
160

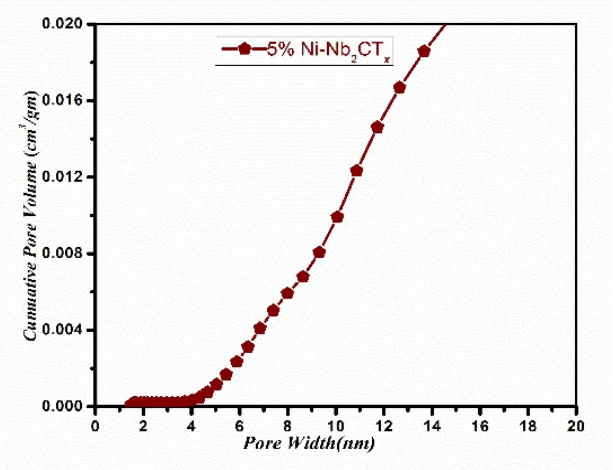
5% Ni-Nb2CTx
Cumuative Pore Volume (cm³/gm)
Pore Width(nm)
0.000
0.004
0.008
0.012
0.016
0.020
0
2
4
6
8
10
12
14
16
18
20

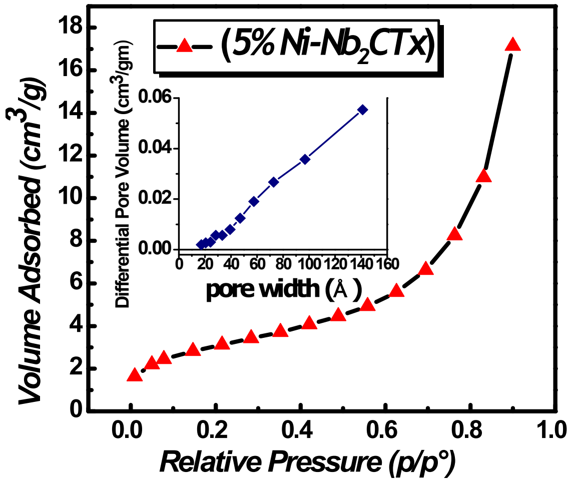

Figure S4: BET Isotherms of MAX and Ni-doped MXene a) 2.5% Ni b) 5% Ni c) 7.5% Ni d) 10% Ni e) differential pore volume VS. Pore width f) cumulative pore volume VS. Pore width

Ni doping %	BET surface area (m^2/g)	Langmuir surface area (m^2/g)	Average particle size (Å)
**0**	5.2149	7.6432	11505.508
**2.5%**	10.9033	15.7189	5502.920
**5%**	17.2017	24.9932	3488.020
**7.5%**	17.9022	25.9701	3351.536
**10%**	18.0221	26.3122	3247.5020

Table S1: BET analysis of MXenes

Fourier Transform Infrared Spectroscopy (FTIR)

Fourier Transform Infrared Spectroscopy (FTIR) spectra of MXene and Ni-doped Nb_2CT_x (Ni =2.5%, 5%, 7.5%, 10%), in the range 500—4500cm^{-1} are shown in Fig. S10. There is a significant shift in all the characteristics peaks towards higher wave number which can be ascribed as the difference in the bond length between Nb, Ni, and C atoms. The peak around 2329 cm^{-1} is considered as the characteristic peak of MXene (Rafiq et al. 2020). The small peak at 2113 cm^{-1} is because of stretching adsorption of C≡C bonds (Luo et al. 2016). Slight humps at 1435 cm^{-1}, 3300 cm^{-1} and 4000 cm^{-1} are for NH_4, N-H and OH groups. The peaks between 550-900 cm^{-1} show typical peaks of Nb-O bands (Castro et al. 2016). Thus, FTIR analysis confirms the efficacious doping of Nickel ions into Niobium Carbide MXene.

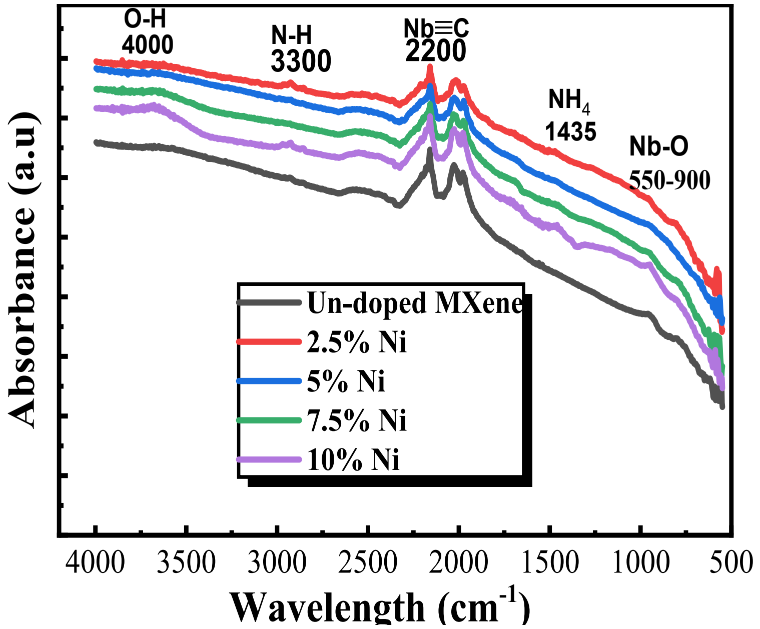

Figure S5: FTIR spectra of pristine MXene and Ni-doped MXene.

References:

1) Castro, Douclasse C., Rodrigo P. Cavalcante, Juliana Jorge, Marco A.U. Martines, Lincoln C.S. Oliveira, Gleison A. Casagrande, and Amilcar Machulek. 2016. "Synthesis and Characterization of Mesoporous Nb2O5 and Its Application for Photocatalytic Degradation of the Herbicide Methylviologen." *Journal of the Brazilian Chemical Society* 27 (2): 303–13. https://doi.org/10.5935/0103-5053.20150244.
2) Luo, Jianmin, Xinyong Tao, Jun Zhang, Yang Xia, Hui Huang, Liyuan Zhang, Yongping Gan, Chu Liang, and Wenkui Zhang. 2016. "Sn4+ Ion Decorated Highly Conductive Ti3C2 MXene: Promising Lithium-Ion Anodes with Enhanced Volumetric Capacity and Cyclic Performance." *ACS Nano* 10 (2): 2491–99. https://doi.org/10.1021/acsnano.5b07333.
3) Rafiq, Sunaina, Saif Ullah Awan, Ren Kui Zheng, Zhenchao Wen, Malika Rani, Deji Akinwande, and Syed Rizwan. 2020. "Novel Room-Temperature Ferromagnetism in Gd-Doped 2-Dimensional Ti3C2Tx MXene Semiconductor for Spintronics." *Journal of Magnetism and Magnetic Materials* 497: 165954. https://doi.org/10.1016/j.jmmm.2019.165954.
